# Long non-coding RNAs CCAT1 and CCAT2 in colorectal liver metastases are tumor-suppressive via MYC interaction and might predict patient outcomes

**DOI:** 10.1371/journal.pone.0286486

**Published:** 2023-06-22

**Authors:** Clemens Franz, Michael Wuehrl, Sibylle Hartmann, Fee Klupp, Thomas Schmidt, Martin Schneider

**Affiliations:** Department of General, Visceral and Transplantation Surgery, University of Heidelberg, Heidelberg, Germany; Universitat des Saarlandes, GERMANY

## Abstract

**Background:**

Liver metastases severely reduce the long term survival of colorectal cancer patients. Long non-coding RNAs (lncRNAs) CCAT1 and CCAT2 have previously been found to be associated with impaired patient outcomes in primary colorectal cancer. We aimed to elucidate the role of CCAT1 and CCAT2 in colorectal liver metastases.

**Methods:**

Total RNA was isolated from 97 human tissue samples of colorectal liver metastases and adjacent normal liver tissue. Gene expression analysis was performed by RT-qPCR and Multiplex ELISA and correlated with patient characteristics and survival. Gene expression, cancer cell migration, invasion, and proliferation were studied after siRNA-mediated knockdown of CCAT1, CCAT2, and MYC in metastatic colorectal cancer cell lines Colo205 and HROC277Met2.

**Results:**

Elevated expression levels of lncRNAs CCAT1 and CCAT2, and their common target MYC in colorectal liver metastases were associated with prolonged progression-free survival after liver resection. High expression of CCAT1 was likewise associated with prolonged overall survival. Knockdown of CCAT1, CCAT2, and MYC resulted in increased migratory and invasive potential in metastatic colorectal cancer cell lines. Gene expression analysis revealed alterations in constituents of Wnt signaling following knockdown.

**Conclusion:**

Our findings demonstrate tumor-suppressive functions of lncRNAs CCAT1 and CCAT2 in colorectal liver metastases. They suppress Wnt signaling directly and indirectly through target gene MYC and might prevent further metastatic spread from colorectal liver metastases.

## Introduction

Long non-coding RNAs (lncRNA) are a broad and heterogeneous group of molecules involved in transcriptional and translational gene regulation. Colon-cancer associated transcript 1 and 2 (CCAT1 and CCAT2) are two lncRNAs first described in primary colorectal cancer. Numerous reports have found a negative association of their expression levels in the primary tumor with patient outcomes [1–3]. Both lncRNAs are located on chromosome 8q24, previously termed a genomic desert due to the absence of coding-genes. However, multiple studies have found that genetic polymorphisms within this region are associated with increased risk for various malignant diseases, including colorectal cancer [4, 5]. Both CCAT1 and CCAT2 upregulate the expression of the MYC proto-oncogene through different mechanisms. CCAT1 prevents miRNA let-7c mediated repression of MYC by binding let-7c. However, MYC itself can increase CCAT1 expression through interaction with its promoter [3, 6]. CCAT2, on the other hand, increases MYC expression through interaction with transcription factor TCF7L2 [7]. The biological functions of CCAT1 and CCAT2 compose numerous complex interactions with miRNAs that have been reviewed elsewhere [8, 9]. One of the main regulators of MYC expression is the canonical Wnt signaling pathway involving beta-Catenin. Alterations of the canonical Wnt pathway are frequently found in many different types of cancer such as the early steps of the adenoma-carcinoma sequence of colorectal cancer [10].

Around 25% of colorectal cancer patients will eventually face a diagnosis of liver metastases. Surgical resection remains the only potentially curative treatment option but is limited by patients’ liver function and the extent of liver involvement [11]. However, recurrence following liver resection is common and more than 50% of patients suffer from recurrence within the first two years, again with the liver as the most commonly involved site [12]. Repeat resection may be a viable option if liver extent is limited [13]. In summary, colorectal liver metastases are common and represent a serious threat to long term survival in colorectal cancer patients. However, the lack of useful predictive biomarkers to monitor disease recurrence limits patient follow-up to imaging and clinical examination. The role of CCAT1 and CCAT2 in liver metastases has remained unknown so far. Our question was whether these two lncRNAs have a functional role in colorectal liver metastases and whether they could represent a diagnostic or prognostic marker.

## Methods

### Patient samples and characteristics

Patient samples were used from the tissue bank of the Surgical Oncology group at the Department of General, Visceral, and Transplantation Surgery at Heidelberg University Hospital. Only patient samples of colorectal liver metastases and corresponding physiologic liver tissue were included in this study. Our patient cohort compromised a total of 97 patients (for patient characteristics see Table 1). All patients had a history of colorectal cancer with synchronous or metachronous hepatic metastases and were subjected to combined resection of the primary colorectal tumour and hepatic metastasis or hepatic metastasis resection alone, respectively. Due to the nature of the German hospital system with a relatively large amount of primary care hospitals, Heidelberg University Hospital receives a large amount of referrals to provide treatment at a tertiary care hospital. Thus, a considerable amount of patients had not been previously received any cancer treatment at our institution prior to the diagnosis of their liver metastasis. All samples had been obtained between 2009 and 2014 and written informed consent was given. This project was approved by the Ethics Committee of the University of Heidelberg (Reference no. S-649/2012 and S-596/2015). Informed consent was obtained from all participants. The study was performed in accordance with the Declaration of Helsinki. A subset of 37 paired samples was randomly selected from this cohort for Multiplex ELISA (for patient characteristics see Table 2). All tissue samples were taken from the surgical specimen by the operating surgeon and handed over to the tissue collection team. All samples were placed into ice-cold sterile D-PBS for transportation into the laboratory. Upon arrival in the laboratory, the tissue was cut into smaller pieces, put into storage tubes and flash-frozen in liquid nitrogen followed by transfer into a -80°C freezer. All clinical information from the electronic patient record were gathered in an internal database. Colorectal cancer tissue identity was confirmed by the pathology report of the Department of Pathology of Heidelberg University Hospital. In order to assess progression-free survival and overall survival, information about patient follow-up was extracted from the electronic health records. In general, patients receive further follow-up at Heidelberg University Hospital following metastasis resection unless they chose a different institution based on their own preference. The calculation of progression-free survival was on imaging findings, i.e., the date of the radiology report raising serious concern for colorectal cancer recurrence. Similarly, information on overall survival was extracted from electronic health record. Only confirmed deaths by Heidelberg University Hospital or the primary care provider were included in this study. Calculation of progression-free survival and overall survival was performed with the logrank test.

**Table 1:** Patient characteristics.

Patient characteristics			n
**Age**	Median	61 years	97
	IQR	53–67 years	
**Gender**	Female	42	97
	Male	55	
**Primary tumor location**	Colon	58	97
	Rectum	39	
**Primary tumor stage**	Unknown	3	97
	T1	5	
	T2	7	
	T3	66	
	T4	16	
**Time to recurrence**	Median	14 months	97
**Metastasis**	Synchronous	56	97
	Metachronous	41	
**Recurrence during follow-up**	No recurrence	41	37
** **	Liver with or without other locations	33	
** **	Outside of liver	23	
**Follow-up**	Median	18.1 months	

**Table 2:** Patient characteristics of subgroup for protein analysis.

Patient characteristics			n
**Age**	Median	61 years	37
	IQR	53–69 years	
**Gender**	Female	19	37
	Male	18	
**Primary tumor location**	Colon	36	37
	Rectum	1	
**Primary tumor stage**	Unknown	2	37
	T1	2	
	T2	2	
	T3	27	
	T4	4	
**Time to recurrence**	Median	9.7 months	37
**Metastasis**	Synchronous	23	37
	Metachronous	14	
**Recurrence during follow-up**	No recurrence	12	37
** **	Liver with or without other locations	16	
** **	Outside of liver	9	
**Follow-up**	Median	20.2 months	

### Real-time PCR

Total RNA was isolated from patient tissue samples and cell culture samples using miRNeasy Mini Kit (QIAGEN, Hilden, Germany) and transcribed into cDNA with RT^2^ First Strand Kit (QIAGEN) for tissue samples and Improm-II-Reverse Transcription System (Promega, Madison, WI, USA) for cell culture samples. Semi-quantitative RT-PCR was performed using Light Cycler ®480 SYBR Green I Master (Roche, Mannheim, Germany) and specific primers (S1 Table). All procedures were performed according to the manufacturer’s instructions. Beta-2-microglobulin was selected as housekeeping gene [14]. Relative expression levels were calculated using 2^-ΔΔCt^-equation [15]. mRNA expression levels in colorectal liver metastases were normalized to their corresponding liver tissue sample.

### Cell culture experiments

Human Colo205 cells derived from malignant ascites of a colorectal cancer patient were purchased from Sigma-Aldrich (Sigma-Aldrich, St. Louis, MI, USA). Human HROC277Met2 cells derived from a colorectal liver metastasis were purchased from CLS Cell Lines Service (CLS Cell Lines Service, Eppelheim, Germany). Colo205 cells were cultured in RPMI with 2 mM Glutamine (Sigma-Aldrich). HROC277Met2 cells were cultured in DMEM/F12 with 3 mM Glutamine (Sigma-Aldrich). All cell culture media were supplemented with 1% Penicillin/Streptomycin (Sigma-Aldrich), and 10% heat-inactivated FCS (Sigma-Aldrich). Cells were grown at 37 °C in a humidified 5% CO2 incubator.

### Transient knockdown of CCAT1, CCAT2, and MYC

Transient knockdown was performed using a pool of siRNA sequences (FlexiTube GeneSolution; Qiagen) targeting human CCAT1, CCAT2, or MYC together with Lipofectamine RNAiMAX (Thermo Fisher Scientific, Waltham, MA, USA). AllStars Negative Control siRNA (Qiagen) served as control. Cells were incubated for 24 hours for RNA extraction and 48 hours for migration and invasion assay, proliferation assay, and protein extraction.

### Migration and invasion assay

Migration and invasion assay was performed by seeding cells onto a 8 μm pore transwell insert (Greiner Bio-One, Kremsmuenster, Austria). The inserts were coated with Matrigel (BD, Franklin Lakes, NJ, USA) for invasion assay. Transfected cells were starved for 18 h in respective cell culture medium without FCS. Afterwards, medium in the lower chamber was replenished with 20% FCS serving as chemoattractant. Invading and migrating cells on the opposite side of the insert membrane were quantified after 24 h by dissolving cell-bound crystal violet (Sigma-Aldrich) in 10% acetic acid (Carl Roth GmbH, Karlsruhe, Germany) and measuring the optical density at 540 nm.

### Proliferation assay

Cellular proliferation following knockdown was assessed using BrdU Cell Proliferation ELISA (Roche) according to the manufacturer’s instructions.

### Western blot

Total protein was isolated from cell culture samples using RIPA lysis buffer with added protease inhibitor (Merck Millipore, Burlington, MA, USA). Primary antibodies against human beta-actin (Cell Signaling Technology, Danvers, MA, USA, 1:2000), histone H3 (Cell Signaling Technology, 1:3000), MYC (Santa Cruz Biotechnology, Dallas, TX, USA; 1:2500), and beta-catenin (abcam, Cambridge, United Kingdom; 1:5000) were incubated overnight at 4° C. After development with an HRP-conjugated secondary antibody (Santa Cruz Biotechnology, 1:1000), semiquantitative analysis was performed with ImageJ software (National Institutes of Health, Bethesda, USA). Densitometric measurement of protein bands was performed. MYC protein expression was normalized to Histone H3, and beta-catenin expression was normalized to beta-actin.

### Multiplex ELISA

Protein was isolated from a subset of human tissue samples described above using MILLIPLEX® MAP Lysis Buffer with added protease inhibitor (Merck Millipore) according to the manufacturer’s instructions. Multiplex protein ELISA was performed using NF-κB Signaling 6-plex Magnetic Bead Kit 96-well Plate Assay (Merck Millipore). Total β-Tubulin Magnetic Bead MAPmates™ (Merck Millipore) were used for normalization of protein expression levels.

## Results

### CCAT1, CCAT2, and MYC expression levels are elevated in colorectal liver metastases

**Fig 1:**
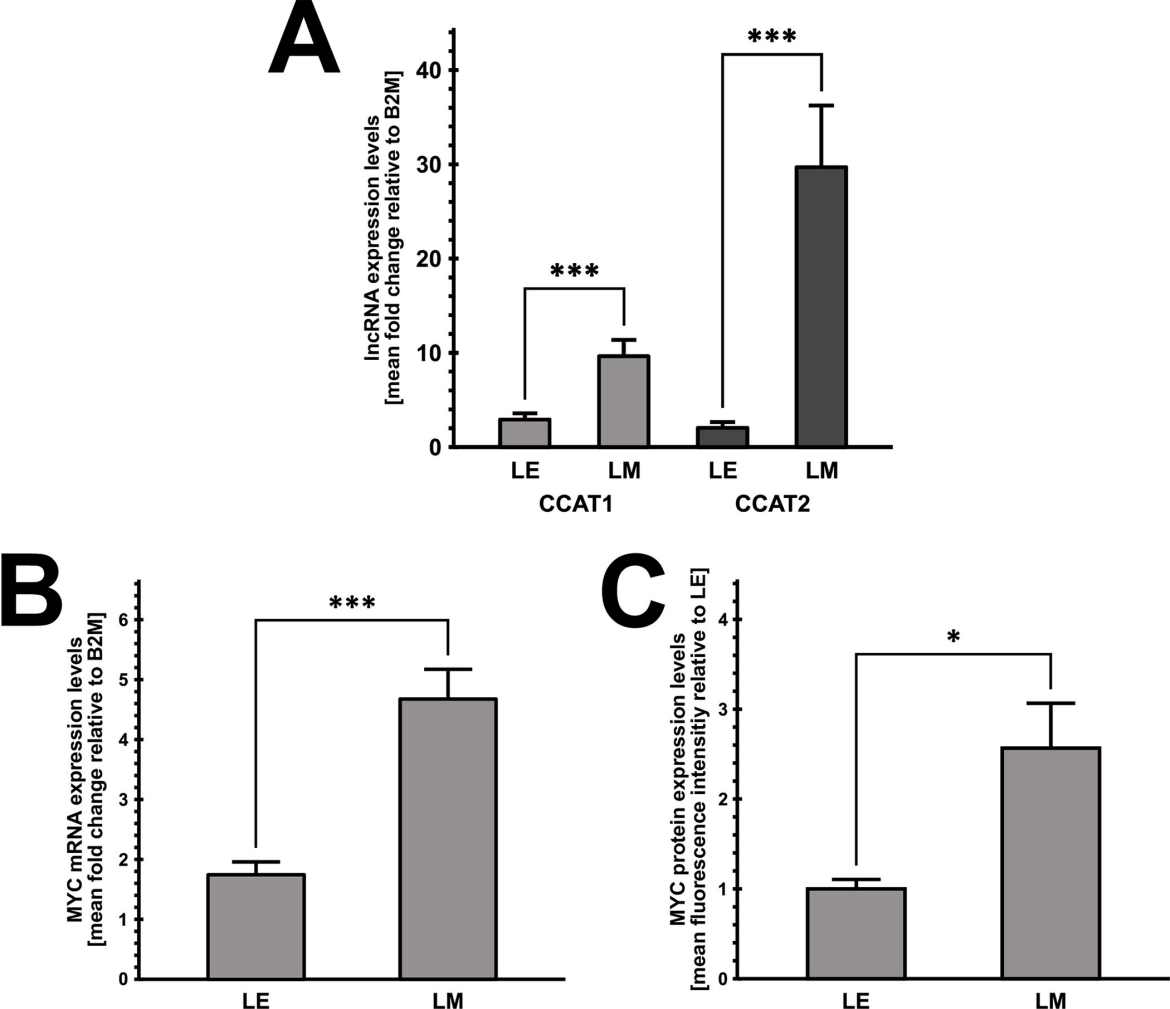
A) Semi-quantitative RT-PCR, revealing significantly upregulated expression of lncRNAs CCAT1 and CCAT2 in colorectal liver metastases (LM) compared to adjacent normal liver tissue (LE) (n = 97, *** p ≤ 0.001, Wilcoxon signed ranks test). B) Semi-quantitative RT-PCR, revealing significantly upregulated MYC mRNA expression in colorectal liver metastases (n = 96, *** p ≤ 0.001, Wilcoxon signed ranks test). C) Multiplex ELISA, revealing significantly upregulated MYC protein expression in colorectal liver metastases (n = 37, * p ≤ 0.05, Wilcoxon signed ranks test).

Gene expression level analysis with semi-quantitative RT-PCR revealed increased expression of lncRNAs CCAT1 and CCAT2 in colorectal liver metastases compared to adjacent liver tissue (Fig 1A). We likewise assessed the expression of their common target MYC. MYC mRNA and protein levels were expectedly elevated in colorectal liver metastases compared to normal liver tissue (Fig 1B and 1C). Correlation of expression of CCAT1 lncRNA with CCAT2 lncRNA and MYC mRNA in liver metastasis was positive with a correlation coefficient of 0.776 (p ≤ 0.001, Spearman’s Rho) and 0.499 (p ≤ 0.001, Spearman’s Rho), respectively. Similarly, correlation of CCAT2 lncRNA with MYC mRNA was positive with a correlation coefficient of 0.283 (p = 0.005, Spearman’s Rho).

### Elevated expression levels of CCAT1 and CCAT2, and their target MYC are associated with beneficial patient outcomes

**Fig 2:**
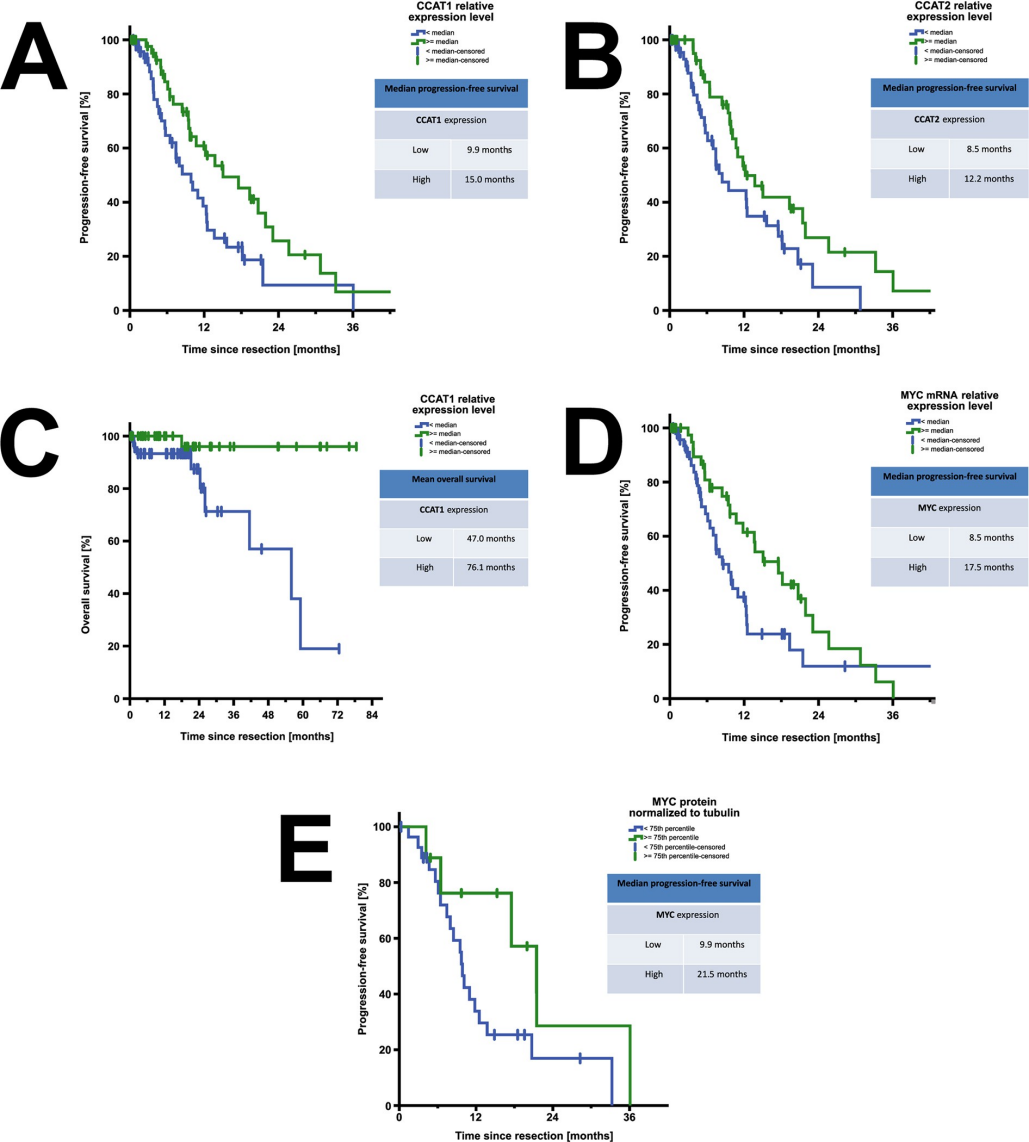
Kaplan–Meier curves revealing oncologic outcomes of patients with low versus high intrametastatic expression of CCAT1, CCAT2 and MYC. A) Elevated relative CCAT1 lncRNA expression levels were significantly associated with increased median progression-free survival (n = 97, p = 0.026, Logrank). B) Elevated relative CCAT2 lncRNA expression levels were significantly associated with increased median progression-free survival (n = 95, p = 0.033, Logrank). C) Elevated relative CCAT1 lncRNA expression levels were significantly associated with increased median overall survival (OS) (n = 97, p = 0.003, Logrank). D) Elevated relative MYC mRNA expression levels were associated with increased median PFS (n = 96, p = 0.018). E) Elevated relative MYC protein expression levels in colorectal liver metastases revealed a trend towards increased median PFS (subgroup analysed by Luminex assay; n = 37, p = 0.092, Logrank).

We next assessed associations of CCAT1, CCAT2 and MYC expression levels in colorectal liver metastases with patient outcomes. For this purpose, we separated our patient cohort into two groups using median lncRNA or mRNA expression as cut-off. Increased expression of lncRNAs CCAT1 and CCAT2 was associated with prolonged progression-free survival following surgical resection of liver metastases (Fig 2A and 2B). Elevated expression levels of CCAT1 were likewise associated with increased overall survival (Fig 2C). Consistently, elevated expression of MYC mRNA and protein was associated with prolonged progression-free survival (Fig 2D and 2E).

### CCAT1 and CCAT2 suppress metastatic cancer cell migration and invasion in vitro

**Fig 3:**
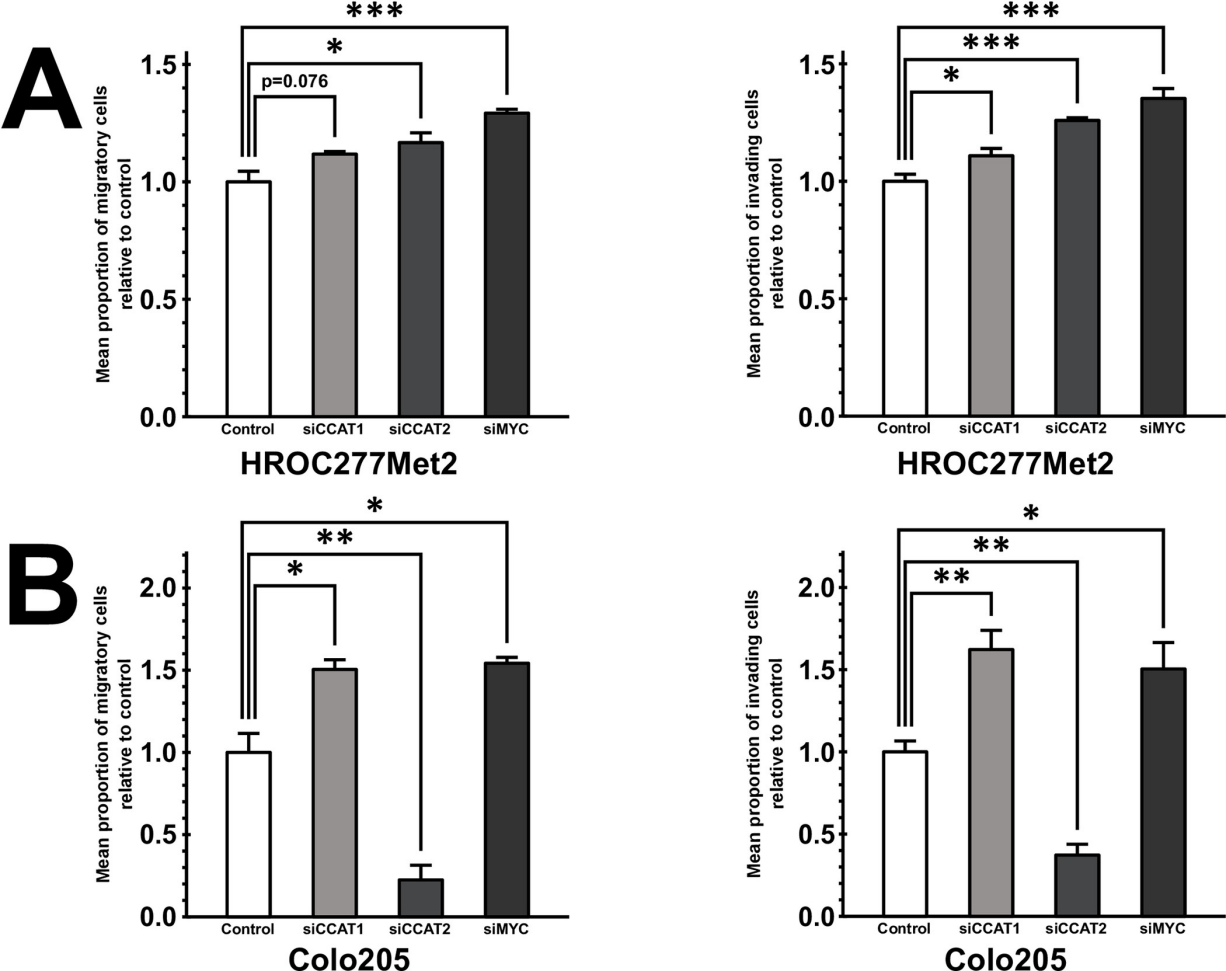
A,B In vitro migration and invasion assays, revealing migratory (left panels) and invasive potential (right panels) of HROC277Met2 (A) and Colo205 cells (B) upon siRNA-mediated knockdown of CCAT1 (siCCAT1), CCAT2 (siCCAT1), and MYC (siMYC), and of control transfected cells (n = 4, * p ≤ 0.05, *** p ≤ 0.001, Student’s t-test).

Based on the clinical observations outlined above we hypothesized that CCAT1 and CCAT2 might exert tumor-suppressive functions in metastatic colorectal cancer cells via their target MYC. We selected two metastatic colorectal cancer cell lines, Colo205 and HROC277Met2 to test this hypothesis (see S2 Table for information on mutational status). Following knockdown of CCAT1 and MYC, we observed an increase in cancer cell migration and invasion in the transwell assay in Colo205 and HROC277Met2 (Fig 3A and 3B).

Knockdown of CCAT2 revealed divergent results among the chosen cell lines. In HROC277Met2, it resulted in a significant increase of migration and invasion (Fig 3A), whereas in Colo205 it prompted strikingly opposite effects (Fig 3B). Remarkably, cellular proliferation was not significantly different between control and knockdowns in all tested cell lines (S1 Fig).

**Fig 4:**
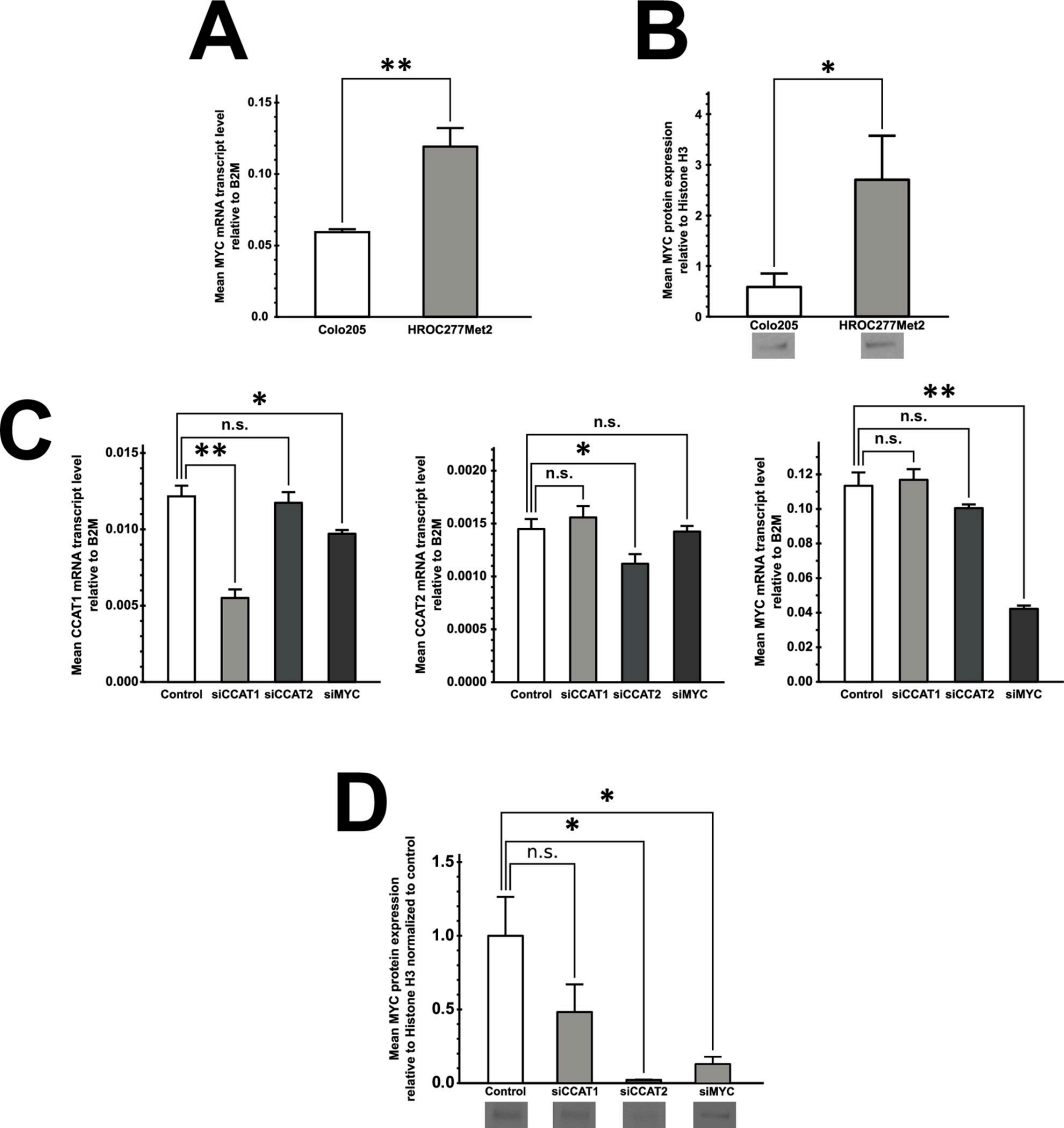
A) Semi-quantitative RT-PCR revealing baseline expression levels of MYC mRNA in HROC277Met2 and Colo205 cells (n = 3, ** p ≤ 0.01, ANOVA). B) Densitometric quantification of Western blot revealing significantly enhanced MYC protein levels in HROC277Met2 compared to Colo205 cells (n = 2 [HROC277Met2] vs. n = 3 [Colo205], * p ≤ 0.05, ANOVA). C) Semi-quantitative RT-PCR revealing expression levels of CCAT1, CCAT2 and MYC in HROC277Met2 cells upon siRNA-mediated knockdown of CCAT1 (siCCAT1), CCAT2 (siCCAT1), and MYC (siMYC), and of control transfected cells (n = 6, * p ≤ 0.05, ** p ≤ 0.01, Student’s t-test). D) Densiometric western blot analysis of MYC protein expression level upon knockdown of CCAT1 (siCCAT1), CCAT2 (siCCAT2), and MYC (siMYC) in HROC277Met2 cells (n = 3, * p ≤ 0.05, Student’s t-test).

Prompted by these findings, we assessed baseline expression levels of MYC in the two studied cell lines. MYC expression was strikingly higher in HROC277Met2 than in Colo205 on both the mRNA transcript and protein level (Fig 4A and 4B). Based on that, we determined that HROC277Met2 represented the more appropriate cell line to further explore putative effects of CCAT1 and CCAT2 on downstream molecular pathways. Overall, knockdown of CCAT1, CCAT2 or MYC revealed divergent patterns of gene response. We initially assessed how knocking down any of these factors affected its respective counterparts. CCAT1 knockdown showed no difference of MYC mRNA expression (Fig 4C, right panel, second bar). In contrast, knockdown of MYC decreased mRNA expression of CCAT1 significantly, consistent with MYC acting as a stimulator of transcription upregulating CCAT1 expression in literature (Fig 4C, left panel, fourth bar). However, knockdown of CCAT1 or CCAT2 resulted in decreased MYC protein expression (Fig 4D).

**Fig 5:**
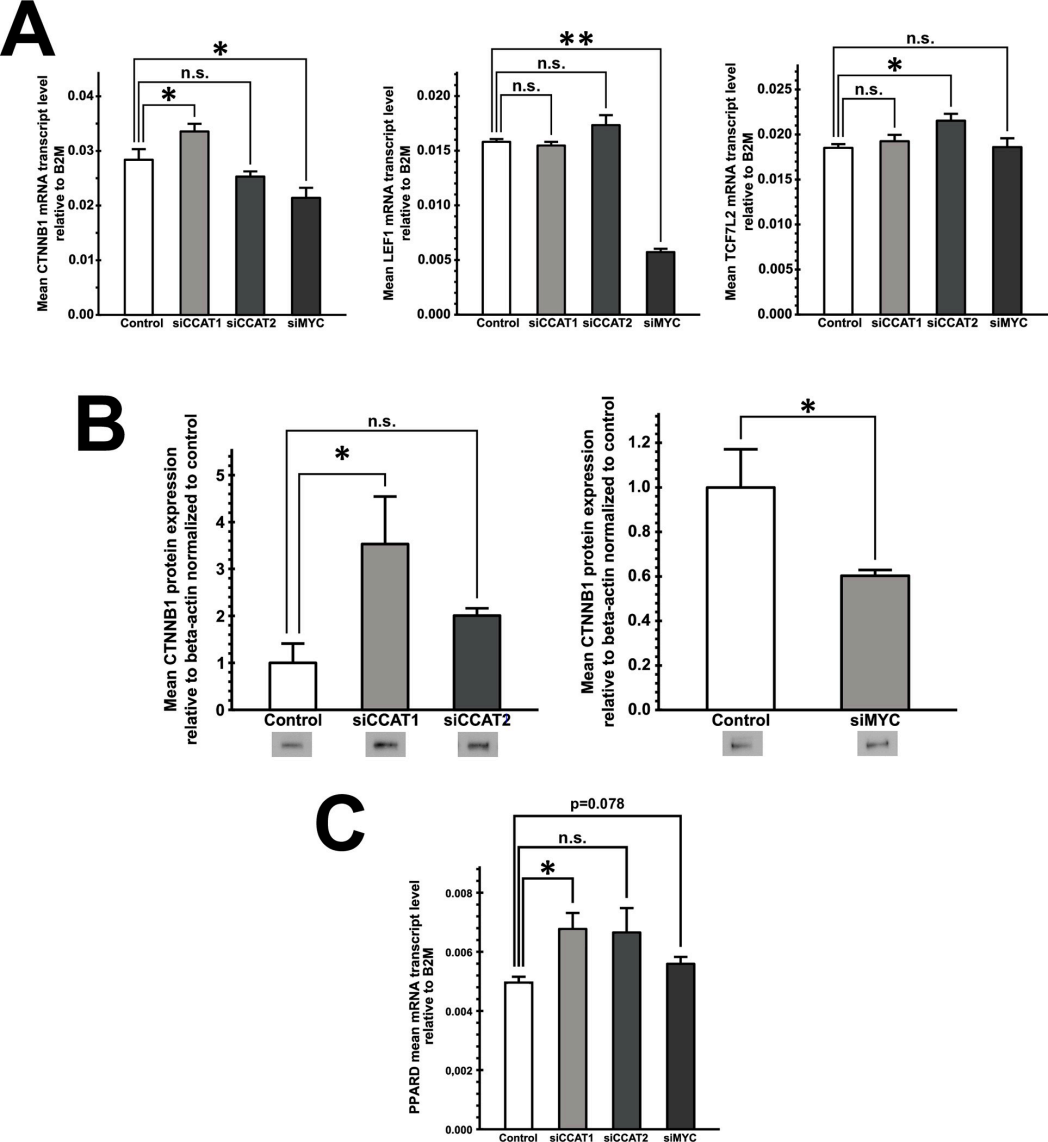
A) Semi-quantitative RT-PCR revealing mRNA expression of Wnt signaling constituents beta-catenin (CTNNB1), TCF7L2 and LEF1 upon knockdown of CCAT1 (siCCAT1), CCAT2 (siCCAT2), and MYC (siMYC) in HROC277Met2 cells (n = 6, * p ≤ 0.05, ** p ≤ 0.01, Mann-Whitney U test). B) Western blot of beta-catenin protein expression upon knockdown of CCAT1, CCAT2 and MYC in HROC277Met2 cells (n = 3, * p ≤ 0.05, Mann-Whitney U test). C) Semi-quantitative RT-PCR of PPARD expression levels upon knockdown of CCAT1, CCAT2 and MYC in HROC277Met2 cells (n = 6, * p ≤ 0.05, Mann-Whitney U test).

We likewise assessed the impact of CCAT1, CCAT2 or MYC knockdown on downstream effectors of Wnt signaling. Surprisingly, MYC knockdown caused a significant decrease of catenin beta-1 (CTNNB1) expression on mRNA and protein levels, despite MYC being a target of Wnt signaling (Fig 5A, left panel; fourth bar, and 5B, bottom panels). On the contrary, CCAT1 knockdown prompted a significant increase of catenin CTNNB1 expression on the mRNA and protein level (Fig 5A, left panel, second bar; and 5B, top panel). CCAT2 knockdown caused no significant change of beta-catenin expression on the mRNA and protein level (Fig 5A, left panel; and 5B, top panels). The transcription factors TCF7L2 and LEF1 are responsible for Wnt signaling mediated gene transcription. MYC knockdown resulted in a significant decrease of LEF1 mRNA levels, whereas CCAT2 knockdown increased TCF7L2 mRNA expression (Fig 5A middle and right panel).

When assessing the impact on Wnt signaling target genes, we found that all three knockdowns increased PPARD mRNA levels, albeit in the case of CCAT2 this was not quite significant (Fig 5C).

Collectively, these findings suggest that CCAT1 knockdown promotes a more invasive and migratory phenotype in vitro, and patients with high CCAT 1 and CCAT2 expression presented with a significantly improved progression free and overall surivial. The altered progression of metastatic colorectal cancer might be mediated via the effects of CCAT1 and CCAT2 on Wnt signaling. These findings might be helpful for stratification of colorectal cancer to improve individualized therapy and follow-up regimens for patients.

## Discussion

Collectively our findings demonstrate tumor-suppressive effects of CCAT1 and CCAT2 in colorectal liver metastases. Elevated expression of both CCAT1 and CCAT2 was associated with increased progression-free survival following resection of colorectal liver metastases. Beyond that, elevated expression of CCAT1 was associated with prolonged overall survival. The association of high MYC expression and increased progression-free survival suggests that these tumor-suppressive functions of CCAT1 and CCAT2 are mediated through their target MYC, a notion that is backed up by further in vitro analyses presented in this study. Elevated expression of CCAT2 and MYC were not associated with a statistically significant increased overall survival. However, this is likely explained by the low mortality in this patient cohort (n = 10).

Cell culture experiments suggest that CCAT1 and MYC propagate a more invasive phenotype in metastatic colorectal cancer cells. Yet, these results are unlikely to be explained by altered proliferation, which was widely unaltered upon knockdown of CCAT1, CCAT2 or MYC. Knockdown of CCAT2 showed opposite results in Colo205 and HROC277Met2. This divergent phenotype could be explained by the different baseline expression levels of MYC in the two cell lines. The biologic output of MYC has been demonstrated to be determined by its baseline expression [16]. Therefore, knockdown of overexpressed MYC in HROC277Met2 could lead to a different phenotype than in Colo205 with its significantly lower expression of MYC. MYC is directly involved in suppressing cancer cell migration and invasion by transcriptional and translational suppression of target genes in a TGF-beta antagonistic manner [17]. In our experiment, knockdown of CCAT1, CCAT2, and MYC resulted in a divergent alteration of gene expression of genes involved in Wnt signaling. However, a uniform increase in transcription of downstream target gene PPARD occurred. Strong evidence supports the role of PPARD in colorectal cancer metastases and was found to be associated with decreased progression-free survival. PPARD downregulation and deletion have proved to suppress cancer cell metastasis in various cancer models in vivo [18]. Interestingly, both PPARD and TGF-beta signaling share a common target, Angiopoetin-like 4, known to be involved in colorectal cancer metastasis [19, 20]. Thus, it seems credible that CCAT1 and CCAT2 suppress these pro-metastatic genes through their interaction with MYC.

Interestingly, MYC expression in HROC277Met2 is attenuated despite knockdown of CCAT1 on mRNA and protein level, whereas CCAT2 knockdown resulted in a significant decrease of MYC protein expression. This may suggest the existence of redundant mechanisms to promote MYC expression in this cell line.

The divergent response of genes involved in Wnt signaling indicates that CCAT1 and CCAT2 possess distinct functions independent of MYC. The increase of TCF7L2 following knockdown of CCAT2 may indicate a regulatory feedback loop that preserves MYC mRNA expression. Furthermore, a trend towards increased beta-catenin protein expression was observed, as well. Similarly, CCAT1 knockdown showed a trend towards increased mRNA and protein expression of beta-catenin. In total, this would suggest increased Wnt signaling following knockdown of CCAT1 and CCAT2. However, previous reports have reported a contrary stimulating effect of CCAT1 on Wnt/beta-catenin signaling in oral squamous cell cancer [21].

To our surprise, MYC knockdown resulted in decreased mRNA expression of beta-catenin and LEF1, despite MYC being a Wnt signaling target itself. This suggests that MYC expression generates a positive feedback loop with Wnt signaling. Furthermore, it has previously been reported that MYC-dependent LEF1 induction can increase beta-catenin expression [22].

From a clinical point of view, CCAT1 and CCAT2 seem to exert protective functions in patients suffering from colorectal liver metastases. However, this should not be confused with a tumor-suppressive function of both lncRNAs but rather represents a different tumor biology. Surprisingly, the roles of CCAT1 and CCAT2 have previously been associated with unfavourable patient outcomes in various tumor diseases including primary colorectal cancer [2, 23–27]. In the light of that our data may appear conflicting, yet our observations could be explained by a functional switch of both lncRNA. Apparently the mechanisms that have promoted metastasis in the primary tumor no longer serve this purpose in the metastasis. The major reason why this is the case may be the different tumor microenvironment. The important role of the tumor microenvironment has been demonstrated in recent years. Interactions may take place both directly or indirectly between host and tumor cells, the immune infiltrate, or the general biomechanical composition [28–30]. From a clinical point of view the main challenge of colorectal liver metastases is recurrence of metastases, either within the liver or in a different location [12]. Yet the expression of CCAT1 and CCAT2 may still beneficial for the liver metastasis, for example immune evasion or growth. A limitation of this study is the lack of an adequate amount of samples from secondary metastases, therefore we do not know in what way CCAT1 and CCAT2 play a role in these entities.

In summary, our study describes the protective effect of CCAT1 and CCAT2 mediated by their target MYC in colorectal liver metastases. Moreover, we were able to demonstrate the direct impact of CCAT1, CCAT2, and MYC on metastatic colorectal cancer cell migration by affecting targets of Wnt signaling, including the upregulation of PPARD. Finally, the divergent response of Wnt genes following knockdown demonstrates the existence of redundant mechanisms of action of CCAT1 and CCAT2 independent of MYC. It has previously been shown that colorectal metastases can seed metastatic cancer cells into the circulation [31]. Based on the observed protective role of CCAT1 and CCAT2 in vivo and the less invasive phenotype in vitro, we conclude that CCAT1 and CCAT2 may suppress metastatic spread originating from colorectal liver metastases. Therefore, we propose CCAT1 and CCAT2 as novel biomarkers to identify patients at higher risk of recurrence who may profit from intensified imaging-based follow-up regimens to timely detect cancer recurrence and initiate therapy. Prospectively collected data in a translational multi-center approach might gain more insight in the process of metastasic spread of colorectal cancer and help to improve individual patient outcome in the future.

## Supporting information

S1 Fig No significant difference in proliferation was found after knockdown of CCAT1, CCAT2, and MYC in both cell lines (n = 2 [HROC277Met2 siCCAT2] vs. n = 3 [all other conditions], ANOVA). (TIF)

S1 Table Primer sequences. (DOCX)

S2 Table Mutation profile of Colo205 and HROC277Met2 cancer cells [32, 33]. (DOCX)
