# Supplementary figures and images for: Long non-coding RNAs CCAT1 and CCAT2 in colorectal liver metastases are tumor-suppressive via MYC interaction and might predict patient outcomes
Source: PLoS One. 2023 Jun 22;18(6):e0286486. doi: 10.1371/journal.pone.0286486 (PMC10287004; doi:10.1371/journal.pone.0286486)

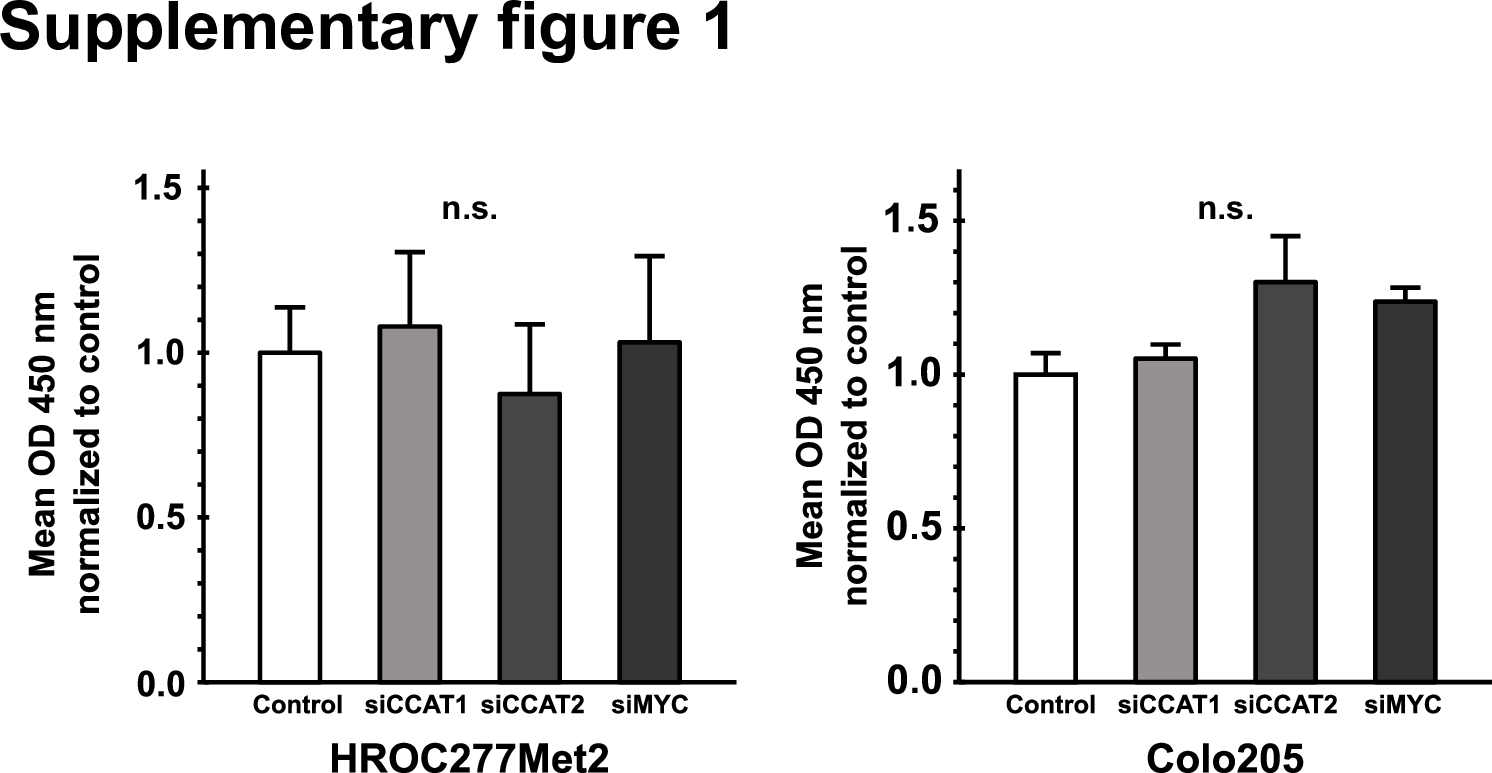

Supplement: S1 Fig — (TIF) [file pone.0286486.s001.tif]
