# Supplementary material for: Long non-coding RNAs CCAT1 and CCAT2 in colorectal liver metastases are tumor-suppressive via MYC interaction and might predict patient outcomes
Source: PLoS One. 2023 Jun 22;18(6):e0286486. doi: 10.1371/journal.pone.0286486 (PMC10287004; doi:10.1371/journal.pone.0286486)
Supplement: S1 Table — (DOCX) [file pone.0286486.s002.docx]

Supplementary table 1 – Primer sequences

| **Gene name** | **Sequence forward primer** | **Sequence reverse primer** |
| --- | --- | --- |
| B2M | TGACTTTGTCACAGCCCAAG | GCAAGCAAGCAGAATTTGG |
| CCAT1 | TCACTGACAACATCGACTTTGAAG | GGAGAAAACGCTTAGCCATACAG |
| CCAT2 | CCCTGGTCAAATTGCTTAACCT | TTATTCGTCCCTCTGTTTTATGGAT |
| CTNNB1 | CCTATGCAGGGGTGGTCAAC | CGACCTGGAAAACGCCATCA |
| LEF1 | GCATCAGGTACAGGTCCAAGA | ACGTTGGGAATGAGCTTCGT |
| PPARD | GCGGACCTGGGGATTAATGG | TGCCCAAAACACTGTACAACAC |
| TCF7L2 | CGCGCGGGATAACTATGGAA | ACAGTGCCCGACACTTCTTT |
