# Supplementary material for: Long non-coding RNAs CCAT1 and CCAT2 in colorectal liver metastases are tumor-suppressive via MYC interaction and might predict patient outcomes
Source: PLoS One. 2023 Jun 22;18(6):e0286486. doi: 10.1371/journal.pone.0286486 (PMC10287004; doi:10.1371/journal.pone.0286486)
Supplement: S2 Table — (DOCX) [file pone.0286486.s003.docx]

Supplementary table 2 – Mutation profile of Colo205 and HROC277Met2 cancer cells [32, 33]

| **Cell Line** | **Mutations per Mb** | **Percentage non-diploid** | **MSI status** | **NSPH status** | **CIMP status** |  |  |  |  |  |
| --- | --- | --- | --- | --- | --- | --- | --- | --- | --- | --- |
| **HROC277Met2** | 9,61 | 54,8 | MSS | Neg | CIMP+ |  |  |  |  |  |
| **COLO205** | 8,47 | 83,5 | MSS | Neg | CIMP+ |  |  |  |  |  |
|  | **MMR pathway** | | | | | | |  |  |  |
| **Cell Line** | MLH1 methylation | MLH1 | MLH3 | MSH2 | MSH3 | MSH6 | PMS2 |  |  |  |
| **COLO205** | Neg | wt | wt | wt | wt | wt | wt |  |  |  |
|  | Wnt pathway | | | | | | | | | |
| **Cell Line** | **APC** | **SOX9** | **BCL9L** | **AXIN2** | **FBXW7** | **FAT1** | **CREBBP** | **CTNNB1** | **TCF7L2** | **RNF43** |
| **COLO205** | mut | wt | wt | wt | wt | wt | wt | wt | wt | wt |
|  | MAPK pathway | | | PI3K pathway | | | p53 pathway | |  |  |
| **Cell Line** | **KRAS** | **BRAF** | **ERBB3** | **PIK3CA** | **PIK3C2B** | **TIAM1** | **TP53** | **ATM** |  |  |
| **COLO205** | wt | mut | wt | wt | wt | wt | mut | wt |  |  |
|  | TGF-beta pathway | | | Chromatin remodeling | | |  |  |  |  |
| **Cell Line** | **SMAD4** | **ACVR2A** | **TGFBR2** | **ARID1A** | **CHD6** | **SRCAP** |  |  |  |  |
| **COLO205** | wt | wt | wt | wt | wt | wt |  |  |  |  |
|  | Histone methylation or acetyation | | | | | | |  |  |  |
| **Cell Line** | **ASH1L** | **EP300** | **EP400** | **MLL2** | **MLL3** | **PRDM2** | **TRRAP** |  |  |  |
| **COLO205** | wt | wt | wt | wt | wt | wt | wt |  |  |  |
|  |  |  |  |  |  |  |  |  |  |  |
| **Cell line** | **MSI status** | **K-ras** | **N-ras** | **H-ras** | **PIK3CA** | **B-Raf** | **APC** | **CTNNB1** | **TCF7L2** |  |
| **Colo205** | MSS | wt | N.A. | N.A. | wt | mut | mut | wt | wt |  |
| **HROC277Met2** | MSS | mut | wt | wt | wt | wt | N.A. | N.A. | N.A. |  |
|  |  |  |  |  |  |  |  |  |  |  |
